# Protective effects of cerium oxide nanoparticles in non-alcoholic fatty liver disease (NAFLD) and carbon tetrachloride-induced liver damage in rats: Study on intestine and liver

**DOI:** 10.1016/j.metop.2021.100151

**Published:** 2021-11-18

**Authors:** Ebrahim Abbasi, Seyed Alireza Vafaei, Nima Naseri, Ali Darini, Masoumeh Taheri Azandaryani, Farhad Kian Ara, Fatemeh Mirzaei

**Affiliations:** a Department of Biochemistry, School of Medicine, Hamadan University of Medical Sciences, Hamadan, Iran; b Department of Biology, Islamic Azad University, Sanandaj Branch, Sanandaj, Iran; c Department of Nanotechnology, Pharmaceutical Sciences Branch, Islamic Azad University (IAUPS), Tehran, Iran; d Department of Neurophysiology Research Center, Hamadan University of Medical Sciences, Hamadan, Iran; e Medical Biology Research Center, Kermanshah University of Medical Sciences, Kermanshah, Iran; f Department of Anatomy, School of Medicine, Hamadan University of Medical Sciences, Hamadan, Iran

**Keywords:** Cerium oxide nanoparticles, Liver injury, Inflammation, Rats, Carbon tetrachloride

## Abstract

**Background and aims:**

Nanoparticles could represent a therapeutic approach for the treatment of various diseases. It has been reported that cerium oxide nanoparticles (CeO_2_ NPs) have potential useful effects. Therefore, we aimed to examine the protective effects of the CeO_2_ NPs in two models of liver injury, non-alcoholic fatty liver disease (NAFLD) and carbon tetrachloride (CCl_4_)-induced liver fibrosis, in rats.

**Methods:**

In this experimental study, male rats were randomly divided into different experimental groups including: Experiment 1; group1: healthy rats received normal saline, 2: CCl_4_ group, 3: CCl_4_ + nanoparticle. Experiment 2; group1: healthy rats received chow diet, 2: NAFLD group, 3: NAFLD + nanoparticle. The oxidative stress markers were determined in the liver and intestine. Tumor necrosis factor-α (TNF-α) levels were measured by ELISA. Histopathological changes of liver and intestine were evaluated by light microspore.

**Results:**

Total antioxidant capacity (TAC) and glutathione (GSH) levels significantly decreased, while malondialdehyde (MDA) and total oxidant status (TOS) were significantly increased in the liver, and intestine of the NAFLD and CCl_4_ group compared with control rats. However, the use of nanoparticles significantly normalized these markers. The levels of the TNF-α were significantly reduced in the nanoparticle group as compared with NAFLD model and CCl_4_-treated rats. CeO_2_ NPs also normalized the liver and intestinal histological changes.

**Conclusions:**

Our finding revealed that CeO_2_ NPs has potential protective effects by increasing antioxidant activity, and reducing inflammation.

## 1 Introduction

Liver is known as the main organ that is involved in the metabolism of macromolecules, excretion and detoxification of circulating agents, synthesis of proteins, and bile acids. Experimental animal models are vital to know the mechanisms responsible for liver diseases. Among the animal models of liver cirrhosis and fibrosis, the most generally used is the carbon tetrachloride (CCl_4_) and high-fat diet (HFD), which closely resembles the histological and hemodynamic features of human disease [1]. HFD is known as a main risk factor for the prevalence of various disorders such as non-alcoholic fatty liver disease (NAFLD), obesity, dyslipidemia, cardiovascular disease (CVD), and diabetes. NAFLD is known as the main form of chronic liver disorders throughout the world [2], considering approximately 24% of the worldwide population with the highest estimates reaching in the Middle East (32%) and in South America (31%) [3]. The pathogenesis of NAFLD is defined in terms of the “two hits”, including lipid accumulation (first hit) and increased oxidative stress, inflammation, mitochondrial dysfunction and lipid peroxidation (second hit) which are mainly are responsible for the onset NAFLD to develop non-alcoholic steatohepatitis (NASH) and liver cirrhosis [4]. On the other hand, hepatotoxins, such as carbon tetrachloride (CCl_4_) which is recognized by variable grade of hepatocyte degeneration and cell death [5,6]. CCl_4_ is a chemical pollutant that has numerous adverse effects on the kidney, liver, blood and heart by elevating lipid peroxidation and generating free radicals [7]. CCl_4,_ as a prominent toxin among the other hepatotoxins, is commonly used to induce experimental animal models that mimic human hepatotoxicity. In the liver, the cytochrome P_450_ enzymes catalyzed CCl_4_ into trichloromethyl radical (CCl_3_^•^), which quickly reacts with oxygen to form trichloromethyl peroxy radical (CCl_3_OO^**•**^), the extremely reactive derivative. Both of these radicals by covalent binding to the cell proteins induce lipid peroxidation and oxidative stress (OS), consequently can lead to liver damage [5,7]. The CCl3^•^ and CCl3OO^•^ mediated lipid peroxidation and is known as a major mechanisms of liver damage induced by carbon tetrachloride [5]. Moreover, CCl_4_ can increase inflammatory markers in the body [8]. Inflammatory cytokines such as tumor necrosis factor-α (TNF-α) worsen pathological progression, and lead to liver fibrosis that complicates the liver treatment [9].

Oxidative stress itself has been confirmed to mediate various cellular responses causing diverse outcomes such as cell growth and apoptosis [10]. Oxidative stress is a consequence of the imbalance between cellular antioxidant capacity and reactive oxygen species (ROS) generation [11]. These free radicals are involved in the etiology of various disorder conditions such as neurodegenerative disorders, cancers, cardiovascular diseases, and aging [12]. Therefore targeting lessening of inflammation and oxidative stress are a beneficial strategy to combat liver injury [13]. Unfortunately, therapeutic strategies designed to relieve liver injury have progressed at a slow pace, perhaps because of the adverse effects of chemical medicines [14,15]. Hence, patients often resort to natural products or new agents as an alternative therapy for their illnesses [[16], [17], [18], [19]].

The nanoparticles administration has been documented as a potential therapeutic because of a better cellular uptake and distribution than other chemical medicines. The cerium oxide nanoparticles (CeO_2_ NPs) are one of the main favourable nanoparticles for anti-inflammatory and antioxidant applications [20]. Cerium has two oxidation states, including Ce^+3^ and Ce^+4^. The beneficial effect is attributed to its capability to mimic superoxide dismutase (SOD), acting as effective ROS and reactive nitrogen species (RNS) scavengers (Ce^+3^ to Ce^+4^) and mimic catalase activity (conversion of H_2_O_2_ into oxygen and water) and peroxidase activity (reducing H_2_O_2_ into hydroxyl radicals). CeO_2_ NPs related to antioxidant activity renders the nanoparticles a precious agent for treatment of oxidative-related disorders [20,21]. Hence, we hypothesize that CeO_2_ NPs can potentially reduce liver injury, inflammation, and oxidative stress in experimental HFD (NAFLD model) and CCl_4_-induced liver fibrosis. CCl_4_ can lead to liver fibrosis, but no insulin resistance, nor obesity, and it is no NAFLD model by itself, which is why we use two different liver animal models. In this experiment, we evaluated the effects of CeO_2_ NPs on liver and intestine by assessing the antioxidant activity, chemical factors and histological changes. The aim of the study was to reveal whether CeO_2_ NPs can prevent oxidative stress, and inflammation in the liver and intestine in NAFLD rats models and CCl_4_-induced liver injury.

## 2 Material and methods

All chemicals agents had analytical grade and were purchased from Sigma-Aldrich (Poole, UK). CeO_2_ NPs were obtained from NanoSany (NanoSany Corporation, Mashhad, Iran).

### 2.1 Animal handling and treatment

Male Wistar rats weighing 170–200 g, and aged 7-week were housed in the animal hosue under standard conditions (60–70% humidity, 25 ± 2 °C and 12 h light/dark cycle). The rats were fed on a standard diet and water. The animals were maintained for 7 days prior to the beginning of the experiments. After adaptation, animals randomly divided into different groups as bellow:

Experiment 1; group1: healthy rats received normal saline, 2: healthy + nanoparticle 3: CCl_4_ group, 4: CCl_4_ group + nanoparticle.

**Fig. 1:**
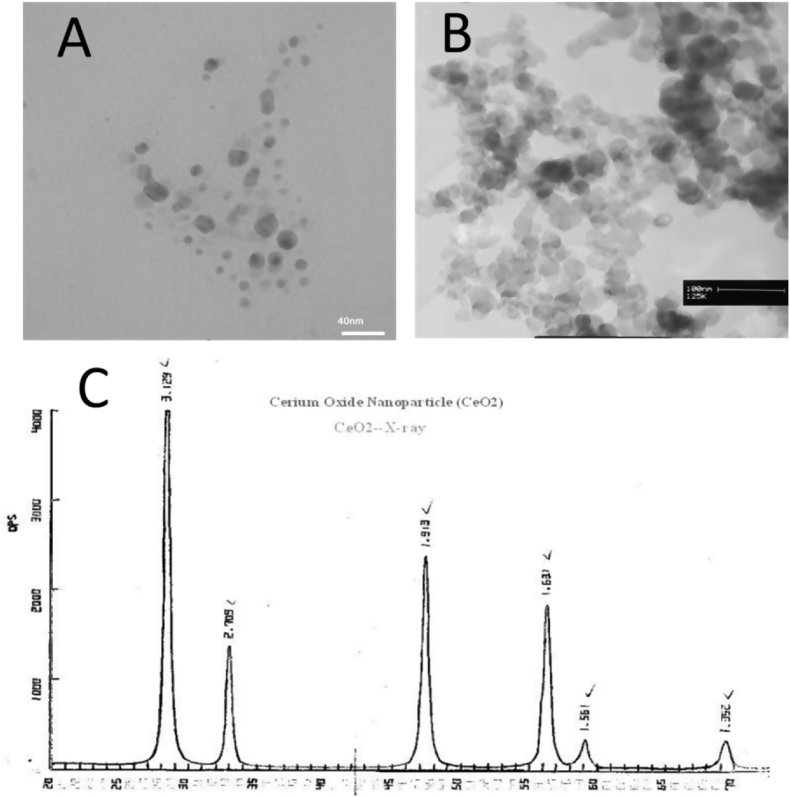
A: Transmission electron microscopy (TEM), B: scanning electron microscope (SEM), and C: X-ray analysis of Cerium Oxide Nanoparticle (CeO_2_, 99.97%, 10–30 nm). NanoSany Corporation, Mashhad, Iran.

Nanoparticle (NanoSany Corporation, Mashhad, Iran. Fig. 1.) was administered for two weeks (0.1 mg/kg, i.v. twice a week for 2 weeks) [22,23], and 2 h after the last administration, liver injury was induced by CCl_4_ (1 ml/kg of 50% CCl_4_, mixture in olive oil, i.p.) [24]. After 24 h, all rats were euthanized (by diethyl ether) and blood samples were collected from the heart.

Experiment 2; group 1: healthy rats, 2: healthy rats + nanoparticle, 3: NAFLD group) 60 kcal% fat), 4: NAFLD group + nanoparticle. Nanoparticle was administered for 4 weeks (0.1 mg/kg, i.v. twice a week). After 24 h, all rats were euthanized (by diethyl ether) and blood samples were collected from the heart.

To achieve serum, the blood sample was allowed to clot and then centrifuged at 3000 g for 10 min. Serum was used for the detection of biochemical tests. Then the animals were sacrificed by cervical dislocation and the liver, and intestine from each animal was removed, and washed in ice-cold saline. Small portion of the liver (left lobe), and intestine (same part for each animal) immersed in liquid N_2_, and stored at −80 °C for antioxidant tests [25]. All steps of this experiment were done in accordance with the Hamadan Medical University ethics committee (Ethic code: IR.UMSHA.REC.1398.520).

### 2.2 Biochemical factors

The serum levels of alkaline phosphatase (ALP), aspartate aminotransferase (AST), alanine aminotransferase (ALT), bilirubin, total protein, albumin, fasting blood sugar, total cholesterol and triglyceride concentrations were measured by colorimetric methods using automated chemical analyzer (Cobas Integra 400 Plus, China).

### 2.3 Protein estimation

About 0.1 g of tissue was homogenized in an 800 μL phosphate-buffered saline (PBS) buffer. The homogenates then were centrifuged to get supernatant, which was used for antioxidant tests.

Protein concentrations were measured by Bradford reagent. Bovine serum albumin was used as the standard [24].

### 2.4 Lipid peroxidation

The concentration of malondialdehyde (MDA), a lipid peroxidation marker, in tissues homogenate was determined by assay of thiobarbituric acid reactive substances (TBARS) formation. The absorbance of the TBARS-MDA complex was read by a spectrophotometer at 532 nm. Results were presented as nmol of MDA/mg protein [26,27].

### 2.5 Total antioxidant activity (TAC)

TAC levels were determined by ferric reducing antioxidant power (FRAP) method. Homogenate samples reduce ferric ions (Fe^3+^) to ferrous (Fe^2+^) in the presence of tripyridyl-*s*-triazine (TPTZ). The absorbance of the blue Fe^2+^-TPTZ complex was measured by a spectrophotometer at 593 nm. The amounts of TAC were expressed as nmol/mg protein [28].

### 2.6 Total oxidative status (TOS)

The total oxidative status (TOS) of the sample was determined by the oxidation of ferrous iron to ferric in the samples. The ferric measurement was performed by xylenol orange. Light intensity was measured by a spectrophotometer at 560 nm [26,27].

### 2.7 Glutathione levels

The level of glutathione (GSH) was determined according to the manufacture instruction (Zellbio, Germany). The amount of GSH was expressed as nmol/mg protein.

### 2.8 Tumor Necrosis Factor-α (TNF-α) levels

The TNF-*α* levels were measured in the serum by ELISA kit according to manufacture instruction (BioLegend, UK). The results were expressed as pg/mg protein.

### 2.9 Histopathological analyses

For morphological evaluation, the liver and intestine samples from different groups were taken and fixed in 10% formalin. Then, fixed samples were embedded in paraffin and cut into 5 μm thick sections. Samples were then stained with haematoxylin and eosin (H & E), and observed by optical microscope. The severity of lesions was classified according to the previous published paper [29].

### 2.10 Statistical analysis

The data were analysed using SPSS 20 software and presented as mean ± standard error of mean (Mean ± SEM). The results were analysed by ANOVA followed by Tukey as post-hoc test. A p value less that 0.05 was assumed significant.

## 3 Results

### 3.1 Body weight

Table 1, Table 2 revealed the body weight of animal groups. The body weight significantly reduced in CCl_4_ -treated rats and increased in NAFLD group. We did not find any significant change in body weight between the hepatotoxic and CeO_2_NPs group, while NPs reduce body weight in the NAFLD group.

**Table 1:** Biochemical factors in different treated animals.

Factors/Groups	Control	Control + CeO_2_ NPs	CCl_4_	CCl_4_ + CeO_2_ NPs
Body weight (gram)	210.50 ± 4.50	220 ± 8	205.30 ± 5.0	208.40 ± 6.50
Liver weight (gram)	5.5 ± 0.4	5.0 ± 0.5	5.9 ± 0.3	5.3 ± 0.35
FBS (mg/dL)	138.20 ± 5.23	134.40 ± 10.7	134.00 ± 10.39	161.40 ± 17.24
Total cholesterol (mg/dL)	69.8 ± 6.25	68.8 ± 3.1	110.00 ± 10.25^#^	70.80 ± 8.62*
Triglyceride (mg/dL)	59.0 ± 4.15	66.4 ± 5.5	121.60 ± 7.71^###^	74.80 ± 7.61**
AST (U/L)	59.8 ± 3.46	48.8 ± 7.5	405.60 ± 33.53^###^	179.80 ± 11.14 ***
ALT (U/L)	84.4 ± 5.22	78.2 ± 5.9	456.20 ± 50.19^###^	312.40 ± 2.12*
ALP(U/L)	112.7 ± 9.95	117.2 ± 10.7	921.00 ± 68.31^###^	681.40 ± 6.14 *
Total protein (g/L)	70.8± 6.87	66.8 ± 4.9	48.40 ± 3.98^###^	68.80 ± 4.76**
Albumin (g/L)	36.60 ± 3.00	32.5 ± 4.3	22.40 ± 1.69^###^	32.40 ± 2.65**
Total bilirubin (mg/dL)	0.29 ± 0.01	0.25 ± 0.03	0.84 ± 0.03^###^	0.36 ± 0.033***
Direct bilirubin (mg/dL)	0.12 ± 0.03	0.12 ± 0.02	0.23 ± 0.03^#^	0.07 ± 0.007*

FBS: fasting blood sugar, AST: aspartate aminotransferase, ALT: alanine aminotransferase, ALP: alkaline phosphatase, CCl_4_: carbon tetrachloride.

**Table 2:** Biochemical factors in different treated animals.

Factors/Groups	Control	Control + CeO_2_ NPs	NAFLD	NAFLD + CeO_2_ NPs
Body weight (gram)	220.50 ± 6.0	230.0 ± 8.0	260.50 ± 15^###^	230.00 ± 10*
Liver weight (gram)	6 ± 1.5	5.5 ± 1.0	13 ± 2.5^###^	6.3 ± 1.4
FBS (mg/dL)	85.60 ± 4.16	70.50 ± 8.5	116.00 ± 4.03^#^	91.40 ± 10.27*
Total cholesterol (mg/dL)	65.0 ± 4.15	88.6 ± 7.8	125.5 ± 7.71^#^	80.0 ± 7.5*
Triglyceride (mg/dL)	75.80 ± 6.25	70.5 ± 7.3	162.60 ± 10.1^###^	76.80 ± 8.62**
AST (U/L)	61.00 ± 2.75	43.40 ± 5.5	143.80 ± 6.31^###^	107.60 ± 7.52***
ALT (U/L)	57.00 ± 5.25	60.0 ± 8.0	118.00 ± 5.71^###^	64.40 ± 5.22***
ALP(U/L)	87.8 ± 6.62	90.6 ± 8.1	135.40 ± 6.49^###^	101.40 ± 9.37*
Total protein (g/L)	70.6± 5.62	66.2 ± 6.7	43.60 ± 3.41^##^	67.80 ± 5.05***
Albumin (g/L)	32.00 ± 2.45	28.2 ± 4.5	20.00 ± 2.0^##^	33.20 ± 3.12**
Total bilirubin (mg/dL)	0.28 ± 0.02	0.22 ± 0.09	0.85 ± 0.35^###^	0.37 ± 0.02***
Direct bilirubin (mg/dL)	0.06 ± 0.03	0.07 ± 0.01	0.32 ± 0.06^###^	0.11 ± 0.02***

FBS: fasting blood sugar, AST: aspartate aminotransferase, ALT: alanine aminotransferase, ALP: alkaline phosphatase, NAFLD: non-alcoholic fatty liver disease.

### 3.2 Blood chemical markers

Table 1, Table 2 exhibit the biochemical factors in different treated animals. The hepatotoxic rats displayed markedly (p < 0.05) higher serum activity of ALP, AST and ALT than the normal group. Pretreatment with nanoparticles significantly normalized ALP, ALT and AST levels in hepatotoxic rats. High levels of liver enzymes induced by HFD and CCl_4_ were alleviated markedly in nanoparticles treatment rats. Furthermore, exposure to CCl_4_ significantly reduced (p < 0.05) total protein, and increased total and direct bilirubin levels. CeO_2_NPs effectively ameliorated these markers as compared with the NAFLD and CCl_4_ group.

Changes in the glucose levels were not significant. Table 1 also reveals a significant (p < 0.05) increase in cholesterol and triglyceride levels in the NAFLD group compared to the control rats. Nanoparticle administration significantly reduced these markers.

### 3.3 Oxidative stress marker

**Fig. 2:**
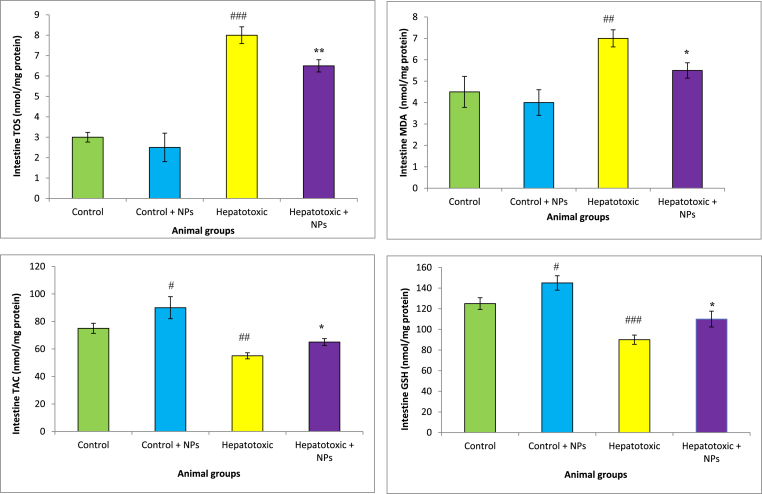
Oxidative stress in the intestine of different treated groups. Results are presented as means ± SEM. *P < 0.05, **P < 0.01, and ***P < 0.001 as compared with Hepatotoxic group (CCl_4_ group). ^###^P < 0.01 compared with control. Results are expressed as means ± SEM of six rats/group. TOS: total oxidant status, TAC: total antioxidant capacity, MDA: malondialdehyde, GSH: glutathione, NPs: nanoparticles.

**Fig. 3:**
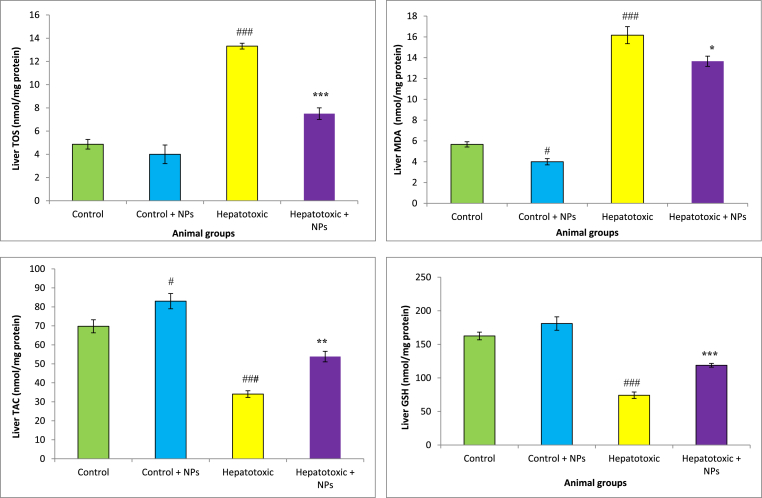
Oxidative stress in the liver of different treated groups. Results are presented as means ± SEM. *P < 0.05, **P < 0.01, and ***P < 0.001 as compared with Hepatotoxic group (CCl_4_ group). ^###^P < 0.01 compared with control. Results are expressed as means ± SEM of six rats/group. TOS: total oxidant status, TAC: total antioxidant capacity, MDA: malondialdehyde, GSH: glutathione, NPs: nanoparticles.

**Fig. 4:**
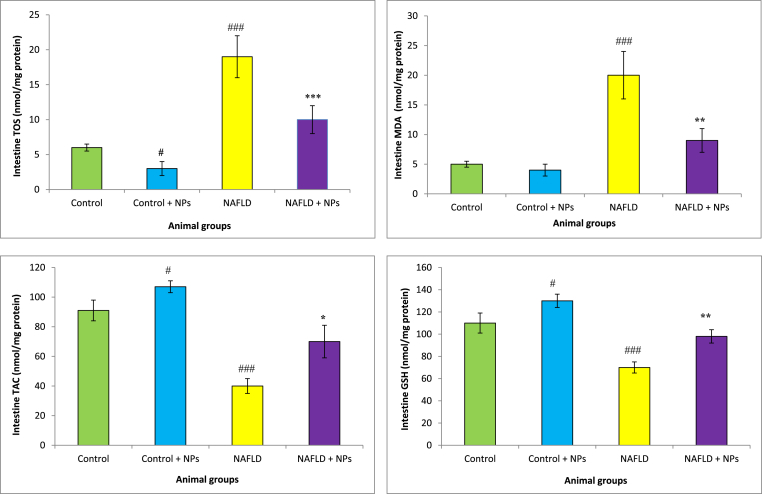
Oxidative stress in the intestine of different treated groups. Results are presented as means ± SEM. *P < 0.05, **P < 0.01, and ***P < 0.001 as compared with Hepatotoxic group (NAFLD group). ^###^P < 0.01 compared with control. Results are expressed as means ± SEM of six rats/group. TOS: total oxidant status, TAC: total antioxidant capacity, MDA: malondialdehyde, GSH: glutathione, NPs: nanoparticles.

**Fig. 5:**
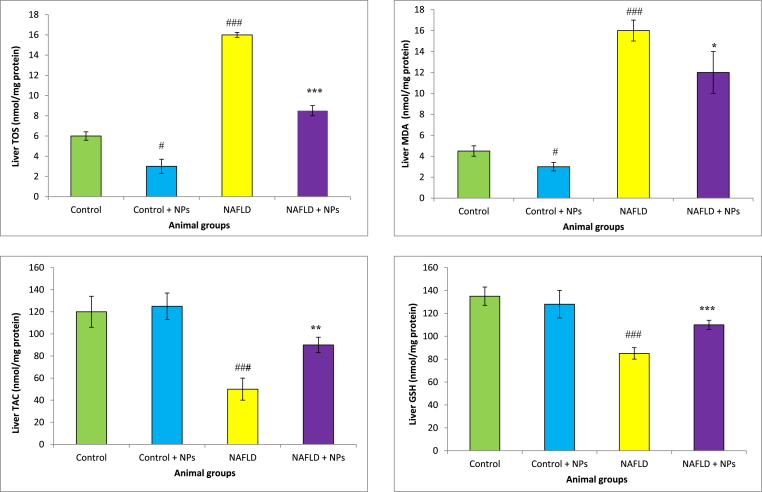
Oxidative stress in the liver of different treated groups. Results are presented as means ± SEM. *P < 0.05, **P < 0.01, and ***P < 0.001 as compared with Hepatotoxic group (NAFLD group). ^###^P < 0.01 compared with control. Results are expressed as means ± SEM of six rats/group. TOS: total oxidant status, TAC: total antioxidant capacity, MDA: malondialdehyde, GSH: glutathione, NPs: nanoparticles.

GSH levels markedly reduced in the liver and intestine of NAFLD group CCl_4_-treated animals compared with the control rats. Administration of CeO_2_NP was increased GSH concentration in these tissues in the hepatotoxic rats. We found a significant rise in TOS in the liver, and intestine of the hepatotoxic and NAFLD group compared with the healthy rats. Furthermore, HFD and CCl_4_ decreased TAC concentration and increased MDA levels in the liver and intestine of animals. Treatment with CeO_2_NPs significantly increased TAC concentrations and reduced MDA levels as compared with the NAFLD and CCl_4_ group (p < 0.01), which showed the mitigation of oxidative stress in these organs (Fig. 2, Fig. 3, Fig. 4, Fig. 5).

### 3.4 TNF-α level

**Fig. 6a:**
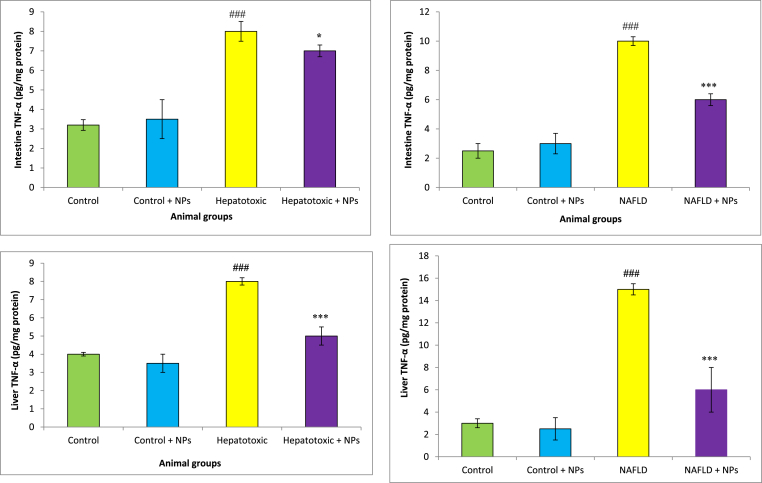
TNF-α levels in different treated groups. Results are presented as means ± SEM. *P < 0.05, **P < 0.01, and ***P < 0.001 as compared with Hepatotoxic group. ^###^P < 0.01 compared with control. Results are expressed as means ± SEM of six rats/group TNF-α: tumor necrosis factor-α, NPs: nanoparticles. b. TNF levels in different treated groups. Results are presented as means ± SEM. *P < 0.05, **P < 0.01, and ***P < 0.001 as compared with NAFLD group. ^###^P < 0.01 compared with control. Results are expressed as means ± SEM of six rats/group TNF-α: tumor necrosis factor-α, NPs: nanoparticles.

The TNF-α level of rats in NAFLD and CCl_4_ groups were significantly higher as compared to control rats (Fig. 6). However, the TNF-α level in the NAFLD and CCl_4_ group which were treated with CeO_2_NPs was obviously (p < 0.05) lower than the untreated group.

### 3.5 Histological alteration

**Fig. 7:**
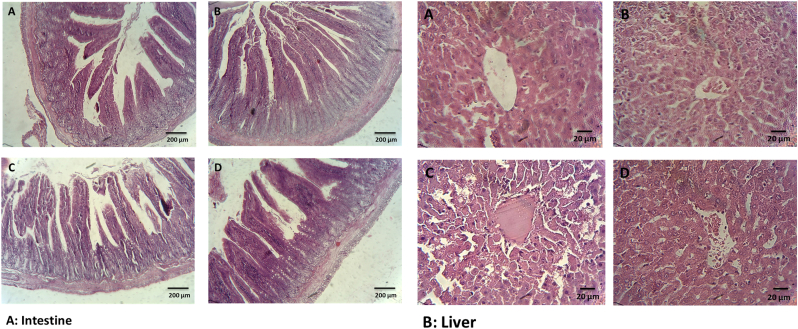
Histological analysis of intestine and liver section in different treated groups stained with H&E. A: healthy rats, B: healthy rats received nanoparticle C: CCl_4_ received rats, D: CCl_4_ received rats received nanoparticle. Liver section of hepatotoxic (CCl_4_) group shows abnormal hepatic structure with vacuolization, necrosis, mild hemorrhage, moderate inflammation, cell hyperplasia, cell infiltration and apoptosis. Liver section of treated animals restored morphological alterations. Original magnification 100 × .

The histological finding supported the liver function test and oxidative stress. Liver samples from healthy group revealed normal lobular structure and cells with a well preserved cytoplasm, and well-defined nucleus without any irregular histological alterations in hepatocytes and lobular architecture (Fig. 7). Liver of NAFLD and CCl_4_–treated rats showed wide liver injuries revealed by moderate necrosis around the central vein, cholangiocyte hyperplasi, hepatic fibrosis, vacuolization and hepatocellular hydropic degeneration. Nonetheless, administration of CeO_2_NPs significantly alleviated the pathological damages.

**Fig. 8:**
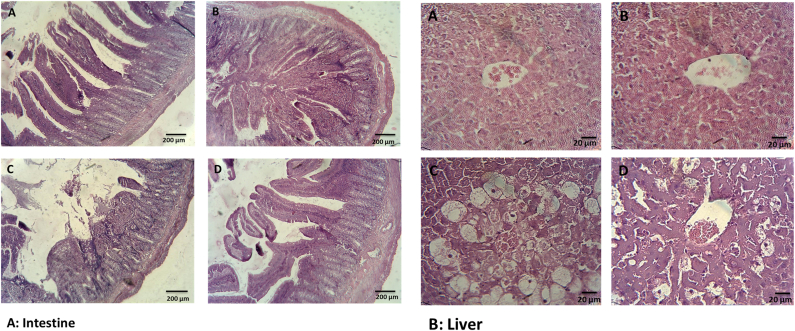
Histological analysis of intestine and liver section in different treated groups stained with H&E. A: healthy rats, B: healthy rats received nanoparticle, C: NAFLD group, D: NAFLD rats received nanoparticle. Furthermore, the NAFLD rats showed hepatic steatosis with ballooning degeneration, inflammatory cell infiltration, and lipid droplet accumulation. Treatment with nanoparticle normalized all of these alterations. Original magnification 400 × .

The histological results revealed mucosal injury, mild edema, disruption, mononuclear cell infiltration, shortening and loosening of intestinal villi, accompanied by spotty hemorrhage, change in the crypt structure, and necrosis in the intestine of NAFLD and CCl4–treated rats.But these alterations were not present in the nanoparticle treated animals (Fig. 8).

## 4 Discussion

In this study we evaluate the hepatoprotective effects of CeO_2_ NPs in the animal models. Previous studies also documented the beneficial effects of CeO_2_ NPs against hepatic oxidative damage caused by the acetaminophen and pyrrolizidine alkaloid monocrotaline [30]. Considering the useful properties of CeO_2_NPs, we further examined whether this nanoparticle may also restore liver functions in NAFLD rats models and CCl_4_ -treated rats. The common dose used in the previous experiment was 0.1 mg/kg [22,23]. In this effective dose, the CeO_2_ NPs decrease oxidative stress, alleviate liver steatosis, and showed anti-inflammatory properties. Therefore, in this study we administered CeO_2_ NPs at the dose of 0.1 mg/kg.

It is well known that HFD substantially alters intestinal physiology and structure. Moreover, HFD promotes intestinal inflammation, oxidative stress and altered barrier integrity [31]. CCl_4_ also can induce oxidative stress in the intestine and increase the production of proinflammatory cytokine and inflammatory cell infiltration in the intestine [32].

In this experiment, the ability of CeO_2_ NPs to protect against HFD and CCl_4_-induced liver injury, oxidative stress and inflammation were examined. Nanoparticles absorption may decrease due to agglomeration/aggregation of the particles in the intestine. Therefore, intravenous administration can be distributed to various organs [33]. In this study, CeO_2_ nanoparticle was administered by intravenous route.

CCl_4_ is a lipophilic agent and is extremely toxic to the hepatocyte [7]. CCl_4_ is metabolized in the liver to generate potentially reactive free radicals and ROS. It is also identified that its oxy metabolite could cause liver toxicity by the depletion of liver GSH. Furthermore, HFD can induce ROS production, accompanied by increased nitric oxide and TNF-α secretion, which promotes chronic inflammation and tissue damage [1]. Our results propose that CeO_2_ NPs can have potential antioxidant properties (by increasing GSH and TAC as well as reducing TOS and MDA).

CCl_4_ intoxication and HFD significantly increased liver enzymes. Liver necrosis causes raises of ALP, AST and ALT levels and an elevated severity of histological hepatic injuries in the animals. In agreement with previous studies [14,15], our result showed a noticeable raise in the ALT, AST, and ALP levels in the hepatotoxic group. CeO_2_ NPs normalized the serum enzymes (ALT, AST and ALP) and led a subsequent recovery towards normalization as compared to the healthy rats, indicating the liver protective effects of CeO_2_ NPs. The noticeable raise in the liver enzyme activity is a sign of the liver injury in the experimental animals [13,34]. Liver is the main organ involved in the blood protein synthesis, particularly albumin. In this experiment, circulating albumin concentration was used to determine liver synthetic ability. The assessment of this protein concentration indicates that CeO_2_ NPs can prevent the reduction of protein likely through neutralizing ROS by scavenger compounds or stabilizing endoplasmic reticulum [35].

Toxic chemicals such as CCl_4_ are oxidized by cytochrome P450 with the following release of liver tissue damaging RNS or ROS resulting in the leakage of liver enzymes into blood. Production of trichloromethyl free radicals (active metabolite of CCl_4_) lead to liver necrosis, malondialdehyde (MDA) production and extracellular matrix destruction. Trichloromethyl radical in the presence of oxygen is converted to trichloromethyl peroxyl radical. These free radicals can covalently bind to protein and membrane lipids to produce MDA, leading to damage to the cells. MDA formation is one of the main reasons of CCl_4_ induced liver and intestine injury. The reduced MDA levels in the liver of the treated-hepatotoxic groups (CCl_4_ and NAFLD), propose the antioxidant and hepatoprotective properties of CeO_2_ NPs [36].

In the present study, marked restorations of glutathione levels and TAC in nanoparticle group were observed when compared with the NAFLD group and CCl_4_ treated groups [37]. TAC is a main defense system against hydroperoxide, ROS, and environmental toxicity. Similarly, glutathione is the first line of defense against oxidative stress [13]. GSH, a main cellular antioxidant, has been recognized as a vital factor needed for liver detoxifications. The depletion of glutathione levels in the liver and intestine may be because of augmented glutathione use in the elimination of trichloromethyl peroxyl radical. Glutathione has a key role in detoxifying the toxic metabolites and liver damage begins when glutathione levels are significantly reduced. Glutathione has long been considered as a key factor in detoxification of the toxic metabolites of CCl_4_.

CCl_4_ and high consumption high fat diets, induce a permanent state of inflammatory cytokines. Inflammation is usually related with liver fibrosis. In this respect, TNF-α is a main factor that induces the inflammatory pathways involved in liver injury [9]. Administration of CeO_2_ NPs significantly reduced TNF-α concentration in different organs as compared to hepatotoxic groups. TNF-α activates the different pathways after liver damage, consequently induces hepatocyte apoptosis, hepatocyte proliferation, and liver inflammation [38].

Liver has a vital role in the metabolism of macronutrients. Administration of CCl_4_ and HFD lead an obvious rise in the total cholesterol (TC) and triglyceride (TG) levels. The disturbance in the phospholipids metabolism and decrease in protein synthesis may result in dyslipidemia. Pretreatment with CeO_2_ NPs modulated blood lipid profiles. It has been reported that CCl_4_ induces the transfer of acetate into hepatocytes and causes rise in cholesterol synthesis. CCl_4_ also elevate the triglyceride synthesis from acetate and increases lipid esterification. The TG accumulation in the liver may happen because of the suppression of lipase activity and secretion of very-low-density lipoprotein (VLDL) [35]. On the basis of our results, a better lipid clearance rate in nanoparticle treated rats was observed.

The hepatoprotective influence of CeO_2_ NPs was further approved by histopathological analyses. High fat diet and CCl_4_ induced liver damages, including necrosis, steatosis, foam cell formation, degeneration of biliary and vacuolization. Nevertheless, in the CeO_2_ NPs treated rats, less degeneration was observed, indicating that this nanoparticle can prevent liver injury or cause the restoration of damaged liver parenchyma [35]. CeO_2_ NPs also normalized histopathological change in the intestine. Our results showed that CeO_2_NPs normalized intestine and liver function by reducing oxidative stress and inflammation. Therefore, this nanoparticle treatment may be consider as a potential agent for liver diseases.

## Availability of data and material

Data are available upon reasonable request.

## Declaration of competing interest

The authors report no conflict of interest.

## CRediT authorship contribution statement

**Ebrahim Abbasi:** Conceptualization, Data curation, Project administration, Supervision, Writing – original draft. **Seyed Alireza Vafaei:** Conceptualization, Methodology. **Nima Naseri:** Project administration, Supervision, Data curation. **Ali Darini:** Data curation, Methodology. **Masoumeh Taheri Azandaryani:** Writing – original draft, Methodology. **Farhad Kian Ara:** Writing – original draft, Methodology, Data curation. **Fatemeh Mirzaei:** Project administration, Methodology, Data curation.
